# Grouper cGAS is a negative regulator of STING-mediated interferon response

**DOI:** 10.3389/fimmu.2023.1092824

**Published:** 2023-02-07

**Authors:** Luhao Zhang, Xin Zhang, Jiaming Liao, Linting Xu, Shaozhu Kang, Hong Chen, Mengshi Sun, Siting Wu, Zhuqing Xu, Shina Wei, Qiwei Qin, Jingguang Wei

**Affiliations:** ^1^ College of Marine Sciences, South China Agricultural University, Guangdong Laboratory for Lingnan Modern Agriculture, Guangzhou, China; ^2^ Guangdong Provincial Key Laboratory of Aquatic Animal Disease Control and Healthy Culture, Zhanjiang, China; ^3^ Laboratory for Marine Biology and Biotechnology, Qingdao, China; ^4^ Pilot National Laboratory for Marine Science and Technology, Qingdao, China; ^5^ Southern Marine Science and Engineering Guangdong Laboratory, Zhuhai, China; ^6^ Department of Biological Sciences, National University of Singapore, Singapore, Singapore

**Keywords:** *Epinephelus coioides*, CGAS, SGIV, virus replication, STING

## Abstract

Cyclic GMP-AMP synthase (cGAS) is one of the classical pattern recognition receptors that recognizes mainly intracytoplasmic DNA. cGAS induces type I IFN responses to the cGAS-STING signaling pathway. To investigate the roles of cGAS-STING signaling pathway in grouper, a cGAS homolog (named EccGAS) was cloned and identified from orange-spotted grouper (*Epinephelus coioides*). The open reading frame (ORF) of EccGAS is 1695 bp, encodes 575 amino acids, and contains a Mab-21 typical structural domain. EccGAS is homologous to *Sebastes umbrosus* and humans at 71.8% and 41.49%, respectively. EccGAS mRNA is abundant in the blood, skin, and gills. It is uniformly distributed in the cytoplasm and colocalized in the endoplasmic reticulum and mitochondria. Silencing of EccGAS inhibited the replication of Singapore grouper iridovirus (SGIV) in grouper spleen (GS) cells and enhanced the expression of interferon-related factors. Furthermore, EccGAS inhibited EcSTING-mediated interferon response and interacted with EcSTING, EcTAK1, EcTBK1, and EcIRF3. These results suggest that EccGAS may be a negative regulator of the cGAS-STING signaling pathway of fish.

## 1 Introduction

Groupers are one of the main economic fishes on the southeast coast of China and Southeast Asia. With the expansion of grouper aquaculture, the pollution in the water and the corresponding disease outbreaks are detrimental, threatening the aquaculture industry. In particular, the emergence of a major viral pathogen, Singapore Grouper Iridovirus (SGIV) has caused huge losses to aquaculture. SGIV belongs to *Iridoviridae*, the frog iridovirus genus (*Ranavirus*), and is a large cytoplasmic DNA virus, and can cause bleeding and swelling of the spleen of the fish. When grouper infected SGIV, the lethality rate can reach > 90% in a week (1).

The innate immune system is the host defense mechanism that plays a critical role against damage caused by microorganisms, pathogens, and other harmful agents (2). When pathogens invade a host, the pattern recognition receptors (PRRs), as part of the host innate immune response, identify pathogen-associated molecular patterns (PAMPs), subsequently activating pro-inflammatory cytokines and interferons through a series of signaling cascades to suppress damage (2). PRRs are broadly classified into five classes: DNA receptors, Toll-like receptors (TLRs), NOD-like receptors, RIG-I-like receptors (RLRs), and C-type lectin receptors (3–5). Among them, DNA receptors are an important class of PRRs that recognize foreign cytoplasmic DNA. The genetic material of many organisms, including viruses, is double-stranded DNA (dsDNA), thus DNA receptors recognize this foreign DNA in the cytoplasm, which is important in the intrinsic host immunity against viruses. Several host proteins have been identified as capable of recognizing double-stranded DNA, including TLR9 (6), Z-nucleic acid binding protein (DAI) (7), DEAD-box helicase (DDX)60 (8), DDX41 (9), Interferon gamma inducible protein 16 (IFI16) (10), and cGAS (cyclic guanosine-adenylate synthase) (11).

A widely recognized DNA receptor, cGAS, has been recognized in recent studies as a member of the nucleotidyltransferase (NTase) family (11). Notably, cGAS recognizes almost all double-stranded DNA as it does not depend on the nucleotide sequence. In mammals, cGAS consists of a nucleotidyltransferase domain and two DNA-binding domains at the N-terminal, the nucleotidyltransferase domain in the middle-conserved fragment, and the C-terminal Mab21 domain (11, 12). When cGAS recognizes foreign DNA, the DNA is attracted by its positive surface charge and zinc finger structure, forming a 2:2 dimer between two cGAS and two dsDNA (12). In turn, cGAS is activated, resulting in its conformational change, and thus promoting the production of cGAMP from ATP and GTP (13). Subsequently, cGAMP binds to the interferon gene-stimulating protein STING, which translocates cGAMP from the endoplasmic reticulum to the Golgi apparatus, and, in turn, recruits TANK-binding kinase 1 (TBK1), phosphorylates IRF3 and nuclear factor-κB (NF-κB) to promote their entry into the nucleus. When IRF3 and NF-κB are translocated from the cytoplasm to the nucleus, IFN-β is expressed, and a large number of inflammatory factors and interleukins are produced (14, 15).

In previous studies, a large number of expressed sequence tags (EST) were found in the transcriptome of the grouper spleen before and after infection with SGIV (14, 15). In this study, a cGAS homolog from orange-spotted grouper (EccGAS) was cloned, and its roles in the innate immune response were investigated. The results will provide new and more effective insights for the prevention and treatment of viral infection.

## 2 Materials and methods

### 2.1 Fish, cells and virus

Juvenile grouper (40-50 g in weight) were obtained from the fishery in Yangjiang City, China. They were stored in a recirculating seawater system at 24-28°C and fed twice daily for two weeks. Then three groupers were randomly selected to detect whether the fish was infected with bacteria or viruses. Twelve tissues were extracted from 6 healthy fish, immediately frozen in liquid nitrogen, and stored at -80°C.

Grouper spleen (GS) cells were constructed in our laboratory and are currently kept in our laboratory. GS cells were grown in Leibovitz L15 medium (Wibco, Waltham, MA, USA) containing 10% fetal bovine serum and placed in a 28°C incubator (14, 15). SGIV was isolated from diseased groupers and cultured as previously described (1, 16). Viral cultures were maintained at -80°C.

### 2.2 Antibodies

Rabbit monoclonal anti-green fluorescent protein (GFP) antibody was purchased from Sigma (Burlington, MA, USA), and mouse monoclonal anti-HA antibody was also purchased from Sigma (Burlington, MA, USA). Rabbit monoclonal β-tubulin antibody was purchased from Proteintech (Rosemont, IL, USA). Polyclonal antibody to SGIV protein MCP was prepared in our laboratory. Horseradish peroxidase-labeled goat anti-rabbit antibody as secondary antibody was purchased from KPL (USA).

### 2.3 Cloning of EccGAS and bioinformatic analysis

Primers used to amplify the open reading frame (ORF) of EccGAS were designed according to the EST sequences of cGAS in the grouper spleen transcriptome (1, 16). The ORF of EccGAS was amplified from the tissue cDNA of healthy grouper. Sequences of EccGAS were analyzed using the BLAST program (http://www.ncbi.nlm.nih.gov/blast), and the conservative domains were predicted using the conservative domain database (https://www.ncbi.nlm.nih.gov/cdd/) of NCBI. SignalP 4.1 was used to predict signal peptides and TMHMM Server V. 2.0 was used to predict transmembrane regions. GeneDoc and Clustal X1.83 were used for amino acid sequences alignment of cGAS, and MEGA version 6.0 was used for phylogenetic tree analysis.

### 2.4 RNA isolation and qRT-PCR

Total RNA was performed using the SV Total RNA Isolation System (Promega, United States) following the manufacturer’s instructions. cDNA synthesis was performed with the ReverTra Ace qPCR RT Kit (Toyobo, Osaka, Japan) according to the manufacturers’ instructions. SYBR^®^ Green Real-Time PCR Master Mix (Toyobo) was used to perform the quantitative real-time PCR (qRT-PCR) in an Applied Biosystems QuantStudio 5 Real-Time PCR System (Thermo Fisher, Waltham, MA, USA), as previously described (17). Briefly, each assay was performed in triplicate with the cycling conditions as follows: 95°C for 1 min for activation, followed by 40 cycles of 95°C for 15 s, 60°C for 15 s, and 72°C for 45 s. The primers of target genes are listed in **Table 1**, and β-actin was used as the internal reference gene. The expression levels were calculated using the 2^–ΔΔCT^ method.

**Table 1:** Primers used for host and viral genes expression analysis.

Primers	Sequences (5´-3´)
IRF3-RT-F	GACAACAAGAACGACCCTGCTAA
IRF3-RT-R	GGGAGTCCGCTTGAAGATAGACA
IRF7-RT-F	CAACACCGGATACAACCAAG
IRF7-RT-R	GTTCTCAACTGCTACATAGGG
ISG15-RT-F	CCTATGACATCAAAGCTGACGAGAC
ISG15-RT-R	GTGCTGTTGGCAGTGACGTTGTAGT
ISG56-RT-F	CAGGCATGGTGGAGTGGAAC
ISG56-RT-R	CTCAAGGTAGTGAACAGCGAGGTA
Viperin-RT-F	TCTGGGTAAATTAGTCCAGTTC
Viperin-RT-R	AGGTGTTGATGACCGAGTTG
IL-1β-RT–PF	AACCTCATCATCGCCACACA
IL-1β-RT-PR	AGTTGCCTCACAACCGAACAC
IL-8-RT-PF	GCCGTCAGTGAAGGGAGTCTAG
IL-8-RT-PR	ATCGCAGTGGGAGTTTGCA
TNFα-RT-F	GTGTCCTGCTGTTTGCTTGGTA
TNFα-RT-R	CAGTGTCCGACTTGATTAGTGCTT
PKR-F	GACCTTGGCTCTGTTGGACC
PKR-R	ATGCTTGGCTTCTTTCTTGT
IFN1-RT-F	GTGTCCTTCCCGAATCATCT
IFN1-RT-R	ACAGCCTGCCTGCTTACAAC
IFN2-RT-F	TACAGCCAGGCGTCCAAAGCATC
IFN2-RT-R	CAGTACAGGAGCGAAGGCCGACA
EccGAS-RT-F	CGGGTTTCATTCTCTCAT
EccGAS-RT-R	AGGCACTCCAGTCTGTGT
Actin-RT-R	TACGAGCTGCCTGACGGACA
Actin-RT-F	GGCTGTGATCTCCTTCTGCA
MCP-RT-F	GCACGCTTCTCTCACCTTCA
MCP-RT-R	AACGGCAACGGGAGCACTA
ICP18-RT-F	ATCGGATCTACGTGGTTGG
ICP18-RT-R	CCGTCGTCGGTGTCTATTC
VP19-RT-F	TCCAAGGGAGAAACTGTAAG
VP19-RT-R	GGGGTAAGCGTGAAGACT

### 2.5 Preparation of antiserum of EccGAS

Primers were designed to amplify the ORF of EccGAS (**Table 1**). The PCR product was digested with BamH I and EcoR I (Takara) and subsequently subcloned into the expression vector pET-B2M. Positive clones were incubated at 37°C, and shaked at 220 rpm in a 150 mL LB medium with 100 mg/mL ampicillin. The vector pET-B2M was used as a negative control. When OD600 of the medium reached 0.6, IPTG inducer (final concentration 0.5 mM) was added, and the culture was shaken at 37°C for 3 h. Recombinant EccGAS fusion proteins (named rEccGAS) were purified. The concentration of purified rEccGAS protein was determined *via* Bradford’s method (18). The purified rEccGAS proteins were then used to immunize the New Zealand white rabbits to obtain polyclonal antibodies against EccGAS according to conventional methods (19). Western blot was used to detect the specificity of the antiserum.

### 2.6 Cell transfection

Cell transfection was performed with Lipofectamine 2000 (Invitrogen, USA) according to the manufacturer’s instructions. Briefly, cells were seeded into plates and changed to serum-free medium after the cell density had spread to 80%. Lipofectamine 2000 and plasmids were diluted with Opti-MEM (Gibco, USA) in two separate sterile tubes. After a 5-min incubation at 25°C, Lipofectamine 2000 with the diluted plasmids were mixed gently and thoroughly. The mixture was then incubated at 25°C for 25 min before being added dropwise to the cells. After 5-6 h, the medium was changed to serum medium to continue the culture (20).

### 2.7 Cell localization analysis

GS cells were seeded into 6-well plates containing coverslips (10 mm×10 mm). When the cell density is appropriate, the plasmids are transfected. After 24 h, cells were washed with phosphate-buffered saline and fixed with 4% paraformaldehyde or methanol for 1 h. Cells were permeabilized with anhydrous ethanol for 15 min at -20°C and then blocked with 2% BAS (ready-to-use) for 2 h at 25°C. Cells were incubated with 1% BSA diluted with rabbit polyclonal anti-EccGAS antibody (1:150) or mouse monoclonal anti-HA antibody (1:150) for 2 h. Cells were incubated for 2 h with 1% BSA diluted with rabbit polyclonal anti-EccGAS antibody (1:150) or mouse monoclonal anti-HA antibody (1:150). Cells were then washed with phosphate-buffered saline. FITC-conjugated goat anti-rabbit or goat anti-mouse antibodies were diluted (1:200) with 1% BSA and incubated for 1 h. Cells were then washed with phosphate-buffered saline and treated with 6-dibutylamino-2-phenylindole (DAPI) for 10 min in the dark, and observed under a fluorescence microscope (Leica, Wetzlar, Germany).

### 2.8 Virus infection assay

Three siRNAs targeting EccGAS mRNA were designed to evaluate the mechanism of EccGAS on SGIV infection in GS cells (**Table 2**). Cells were transfected with the same volume of siRNA or control. The cells were infected with SGIV 24 hours later. The cells were then collected at 24 h and 36 h after SGIV infection. The expression levels of SGIV ICP18, VP19, and MCP were analyzed using qRT-PCR (**Table 1**). The expression of SGIV MCP protein was analyzed using western blotting.

**Table 2:** Primers used for silencing EccGAS.

Primers	Sequences (5´-3´)
NC-F	UUCUUCGAACGUGUCACGUTT
NC-R	ACGUGACACGUUCGGAGAATT
EccGAS-siRNA-174-F	GGAGAAGCCGUCUCUUCAATT
EccGAS-siRNA-174-R	UUGAAGAGACGGCUUCUCCTT
EccGAS-siRNA-1042-F	GCAGUGACCCUGACCACAATT
EccGAS-siRNA-1042-R	UUGUGGUCAGGGUCACUGCTT
EccGAS-siRNA-1191-F	GCGAAUGCCGUAUUAUCUUTT
EccGAS-siRNA-1191-R	AAGAUAAUACGGCAUUCGCTT

### 2.9 Dual-luciferase reporter assay

GS cells were plated in 24-well plates. When the cell density is appropriate, a total of 200 ng of zebrafish IFN1-LUc, human NF-κB-Luc, or human ISRE-Luc plasmids were co-transfected with 30 ng of the internal control PRL-sv40 reninase vectors and 600 ng of the target plasmids. After 36 h, cells were collected, and the luciferase activity was detect by the Dual-Luciferase^®^ Reporter Assay System (Promega).

### 2.10 Co-immunoprecipitation assays

GS cells were passaged into cell culture dishes (10cm×10cm). When the cell density is appropriate, co-transfected with relevant the target plasmids. After 36 h, cells were harvested and lysed in RIPA buffer containing protease and phosphatase inhibitors. Samples were processed using the Double Bead™ Protein G Immunoprecipitation Kit (Invitrogen).

10% SDS-PAGE was used to separate immunoprecipitates or whole cell extracts. Separated immunoprecipitates or whole cell extracts were then transferred to Immobilon-P polyvinylidene difluoride membranes (Millipore, St. Louis, MO, USA). The membranes were blocked in 5% skim milk incubated with antibodies for 2 h at 25°C or overnight at 4 °C, washed 3 times with PBST, and incubated with secondary antibodies for 1 h at 25°C. After washing three times with PBST, the immunoreactive protein was visualized by an enhanced chemiluminescence detection kit (Bio-Rad, Irvine, CA, USA). The band intensity was calculated using Quantity-one software (21).

### 2.11 Statistical analysis

GraphPad Prism (version 8.0.2) was used to perform statistical analysis. Data analysis results are shown as mean ± standard error of the mean of three independent experiments. The statistically significant differences were evaluated by the T-Test with a P value (*P < 0.05 and **P < 0.01).

## 3 Results

### 3.1 Amplification and sequence analysis of cGAS gene in grouper

The ORF of grouper cGAS (EccGAS) was 1695 bp in length, encoding 575 amino acids, with a molecular weight of approximately 63.05 kDa and an isoelectric point of 9.47. SMART analysis revealed that EccGAS contains the Mab-21 typical structural domain, while the signal peptide and transmembrane region were absent.

**Figure 1:**
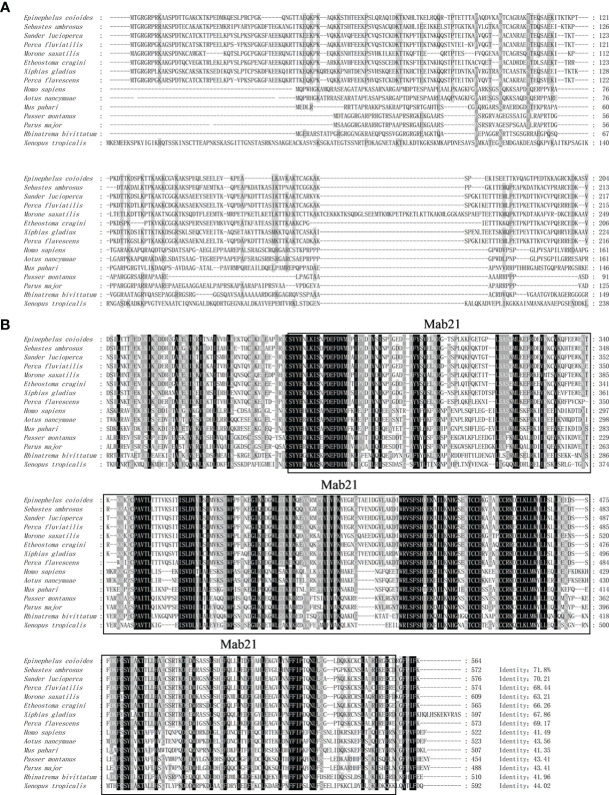
Sequence analysis and phylogenetic tree construction of EccGAS. **(A)** Multiple sequence comparison of the amino acid of EccGAS and cGAS proteins from other organisms. The predicted conserved structural domain of Mab21 is shown. **(B)** Phylogenetic tree of EccGAS proteins. The numbers on the nodes indicate bootstrap values for 1000 replicates. Scale bars represent 0.1 change per site.

Aligning the cGAS proteins of other organisms with EccGAS revealed that the sequences of each species were relatively conserved in the Mab-21 typical structural domain, indicating that cGAS may have similar functions. The cGAS sequences of 11 species were selected for homology comparison, and the multiple sequence alignments were performed with Clustal X (**Figure 1A**). The phylogenetic tree showed that the EccGAS and cGAS of *Sebastes umbrosus* (XP_037637334.1) were more closely related and clustered together (**Figure 1B**).

**Figure 2:**
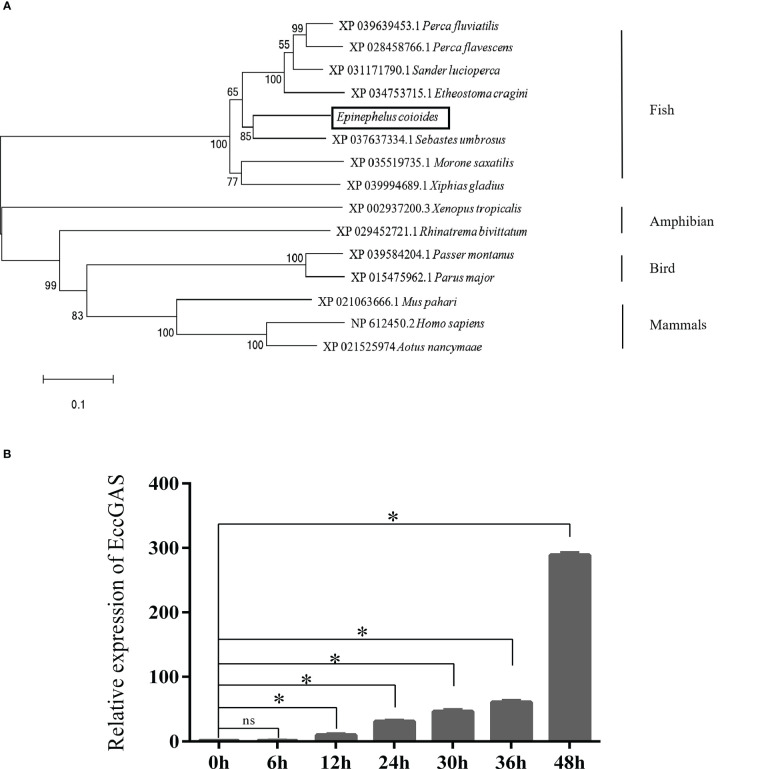
Expression pattern analysis of EccGAS. **(A)** Expression of EccGAS in different tissues of healthy groupers. Data were expressed as a ratio of the tissue value to the EccGAS mRNA expression in liver. β-actin were used as the internal control for the normalization across tissues. **(B)** Expression profiles of EccGAS in GS cells after SGIV infection. β-actin was used as an internal control. *P < 0.05. ns means no significance.

The expression of EccGAS in healthy grouper tissues showed that EccGAS was expressed in all exacted tissues, with higher expression in blood, skin, and gills (**Figure 2A**). After SGIV infection, the transcriptional expression level of EccGAS in GS cells gradually increased and peaked at 36 h.p.i (**Figure 2B**), suggesting that EccGAS may play an important role in the activation of host antiviral innate immunity.

### 3.2 Bioassay of recombinant protein of EccGAS

**Figure 3:**
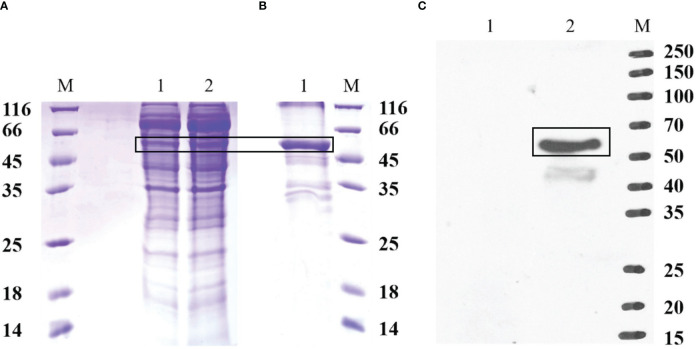
Production of recombinant EccGAS and anti-EccGAS serum. **(A)** Expression of recombinant EccGAS. M: protein molecular quality standard; 1: supernatant of pET-EccGAS induced by sonication; 2: microspheres of pET-EccGAS induced by sonication. **(B)** Purification of recombinant EccGAS. M: protein molecular quality standard; 1:purified recombinant pET-EccGAS protein. **(C)** Preparation of anti-EccGAS serum. M: protein molecular quality standard; 1: purified recombinant pET-EccGAS protein incubated with preimmune mouse serum; 2: purified recombinant pET-EccGAS protein incubated with anti-EccGAS serum (1:5000). Bands of EcCGA protein were boxed.

The recombinant protein of EccGAS was analyzed by SDS-PAGE. A band of 57 kDa was visible using Komas Brilliant Blue staining (**Figure 3A**). The fused recombinant EccGAS protein (rEccGAS) was purified by affinity chromatography with nickel-nitrilotriacetic acid-agarose (QIAGEN, Germany) according to the manufacturer’s instructions (**Figure 3B**). Immunosera were then prepared by immunizing rabbits with rEccGAS protein. rEccGAS protein was specifically recognized by the EccGAS polyclonal antibody, and no bands were detected in the negative control (**Figure 3C**), indicating that the anti-EccGAS antibody specifically recognized the purified EccGAS protein.

### 3.3 Intracellular localization of EccGAS

**Figure 4:**
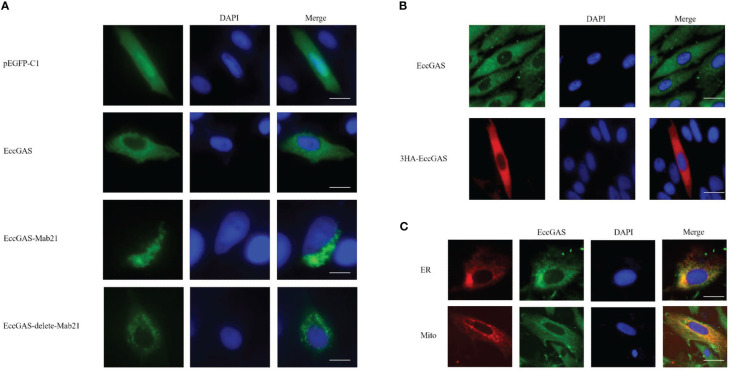
Cellular localization of EccGAS. **(A)** Subcellular localization of the EccGAS structural domain Mab21 and the deletion of the structural domain in GS cells. GS cells were transfected with pEGFP-C1, pEGFP-EccGAS, pEGFP-EccGAS-Mab21 and pEGFP-EccGAS-delete-Mab21 plasmids using Liposome 2000. After transfection for 24 h, the cells were fixed with 4% paraformaldehyde for 2 h at 25 °C, and were stained with DAPI and examined under confocal laser scanning microscopy. Scale bars shown as 20 µm. **(B, C)** Immunofluorescence and colocalization with endoplasmic reticulum and mitochondria. GS cells were inoculated and transfected with pcDNA3.1-EccGAS or pDsRed2-ER and pDsRed2-Mito, primary antibody with rabbit polyclonal anti-EccGAS antibody (1:150) or mouse monoclonal anti-HA antibody (1:150) incubated for 2 h at 25 °C and secondary antibody with FITC-conjugated goat anti-rabbit or goat anti-mouse (1:200). Subsequently, the cells were incubated for 1 h and examined by confocal microscopy. Scale bars are shown as 20 µm.

The expression vectors for full-length EccGAS, structural domain Mab21, and removal of the structural domain were constructed and named EccGAS(EccGAS), EccGAS-Mab21, and EccGAS-delete-mab21. Subsequently, the plasmids were transfected into GS cells, and intracellular localization of EccGAS was determined. The results showed that the green fluorescence of pEGFP-C1 was distributed in the cytoplasm and nucleus of GS cells, while the pEGFP-EccGAS and pEGFP-EccGAS-delete-mab21 were distributed uniformly in the cytoplasm, and pEGFP-EccGAS-Mab21 was distributed in the cytoplasm in an aggregated manner (**Figure 4A**). We next examined the intracellular localization of EccGAS in GS cells by immunofluorescence assay with anti-EccGAS serum. In GS cells, the green and red fluorescences of EccGAS were mainly localized in the cytoplasm by anti-EccGAS serum (**Figure 4B**), which is consistent with the results of subcellular localization.

To explore whether EccGAS co-localizes with organelles, plasmids pEGFP-EccGAS were co-transfected with pDsRed2-ER (endoplasmic reticulum) or pDsRed2-Mito (mitochondria) into GS cells and their localization was examined. The results showed that EccGAS partially co-localized with the endoplasmic reticulum and mitochondria (**Figure 4C**).

### 3.4 EccGAS affected SGIV replication

**Figure 5:**
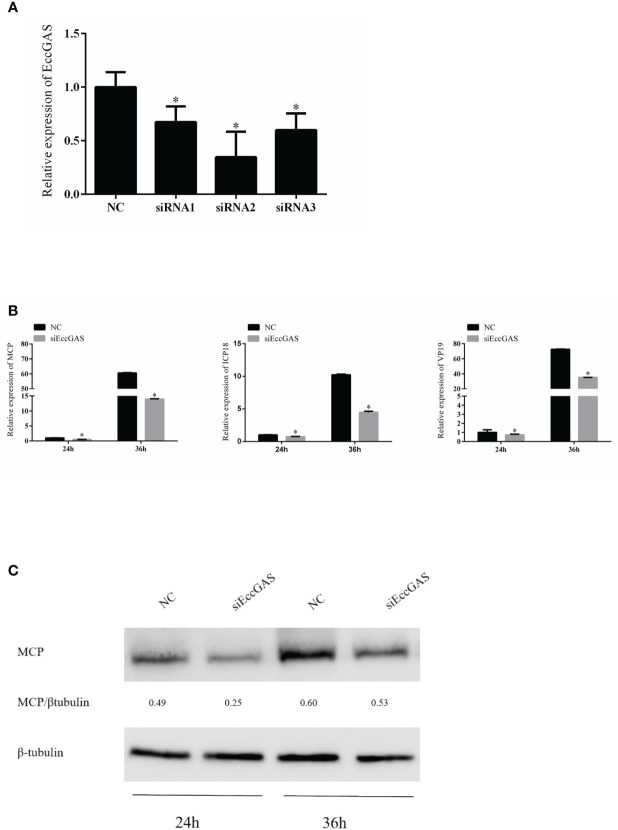
Effect of EccGAS silencing on viral replication. **(A)** Three siRNA sequences were designed based on the sequence of EccGAS and assayed for interference effects. **(B)** EccGAS knockout and control cells were infected with SGIV and collected at 24 h and 36 h to measure the relative expression levels of the viral genes. Relative expression levels of the viral genes were measured by qRT-PCR (n = 3, mean ± SD). *P < 0.05. **(C)** Silencing of EccGAS in GS cells infected with SGIV and protein samples collected for 24 h and 36 h. Expression of MCP protein was detected by western blotting. β-tubulin was used as an internal control. Quantity-one software was used to calculate band intensities and assess the MCP/β-tubulin ratio.

We designed three siRNAs based on the ORF sequence of EccGAS. siRNAs were transfected into GS cells, and qRT-PCR was used to detect the expression of endogenous EccGAS after 24 hours. The results showed that all three siRNAs silenced the expression of endogenous EccGAS in GS cells, of which siRNA2 had the highest interference efficiency (**Figure 5A**). Next, we used siRNA2 to conduct the following experiments. After siRNA2 was transfected into GS cells and infected with SGIV, the expression of SGIV MCP, SGIV ICP18, and SGIV VP19 were examined by qRT-PCR. The expression of SGIV MCP protein was detected by western blot. The results showed that EccGAS knockdown by siRNA2 significantly inhibited the transcript expression of SGIV genes (**Figure 5B**). Moreover, the results of western blot showed that knockdown EccGAS by siRNA2 significantly reduced the expression of MCP protein (**Figure 5C**). These results suggest that EccGAS may promote SGIV replication in GS cells.

### 3.5 EccGAS inhibited interferon immune response

**Figure 6:**
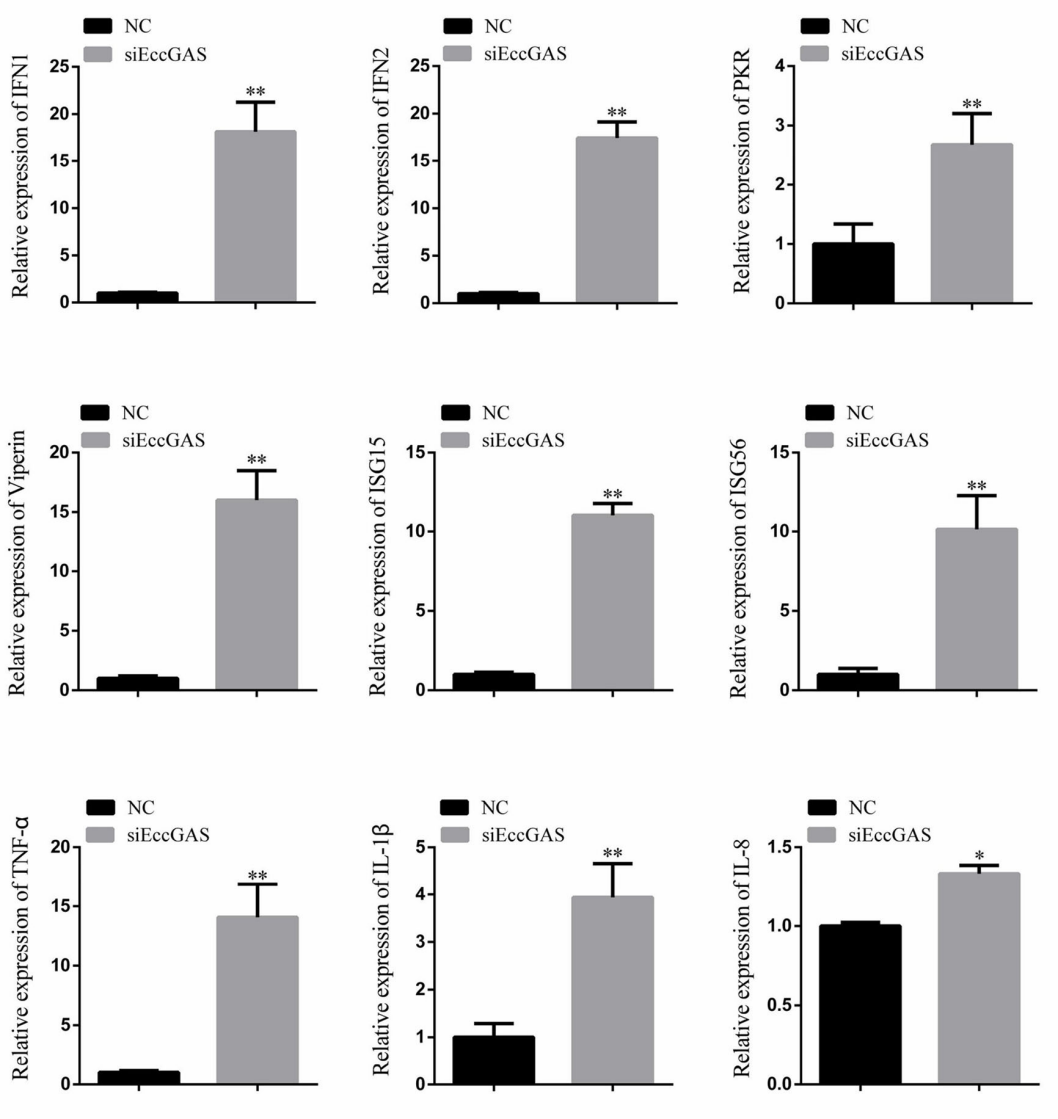
Silencing of EccGAS promoted the expression of host interferon-related genes and inflammation-related factors. Data are expressed as ratio to the control group. Error bars represent mean ± SD; *P < 0.05; **P < 0.01.

The regulatory effects of EccGAS on host immune factors were evaluated by qRT-PCR. As shown in **Figure 6**, EccGAS knockdown by siRNA2 potentiated the transcription of IFN1, IFN2, PKR, Viperin, ISG15, ISG56, IL-1β, IL-8, and TNFα in GS cells. Thus, the results suggested that EccGAS negatively regulated the interferon immune response *in vitro*.

### 3.6 EccGAS inhibits EcSTING-mediated interferon immune response

**Figure 7:**
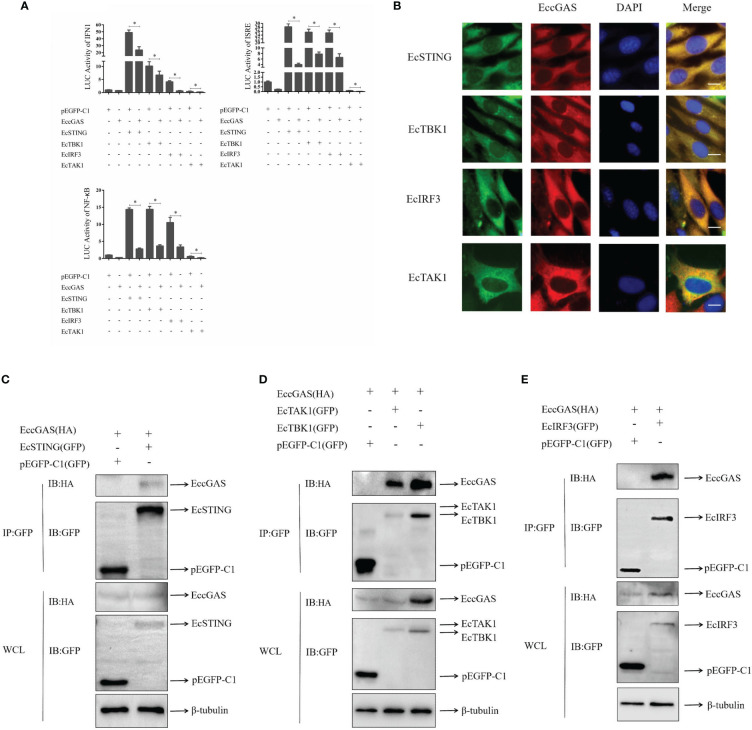
EccGAS negatively regulates EcSTING-mediated immune responses. **(A)** EccGAS inhibits the activation of IFN1, ISRE, and NF-κB promoter induced by EcSTING, EcTBK1, EcTAK1 and EcIRF3. pEGFP-EcSTING, pEGFP-EcTBK1, pEGFP-EcTAK1, and pEGFP-EcIRF3 were transfected into GS cells at a ratio of 1:1:1 with pEGFP-EcGAS and IFN1-Luc, respectively. Cells were harvested for detection of luciferase activity at 36 h after transfection.The error bars indicate mean ± SD; *P < 0.05. ISRE and NF-κB promoter activities were tested in the same way. **(B)** EccGAS colocalized with EcSTING, EcTBK1, EcTAK1 and EcIRF3, respectively. The plasmid pcDNA3.1-Red-EccGAS was co-transfected with pEGFP-EcTBK1, pEGFP-EcTAK1 and pEGFP-EcIRF3 in GS cells, respectively. Imaged by confocal microscopy, scale bars are shown at 20 μm. **(C–E)** EccGAS interacts with EcSTING, EcTBK1, EcTAK1 and EcIRF3, respectively. pEGFP-C1, pEGFP -EcSTING, pEGFP-EcTAK1, pEGFP-EcTBK1, and pEGFP-EcIRF3 were cotransfected with pcDNA3.1-EccGAS in GS cells, respectively. Samples were processed by immunoprecipitation and western blotting. β-tubulin was used as an internal control.

In mammals, cGAS has been reported to produce cGAMP after DNA sensing to stimulate STING, which in turn activates interferon production and innate immunity. We aimed to determine whether EccGAS is involved in EcSTING-mediated interferon immune response. As shown in **Figure 7A**, when EccGAS was co-transfected with EcSTING, EcTBK1, EcTAK1, and EcIRF3, the activities of IFN1, ISRE, and NF-κB promoters were significantly reduced, suggesting that EccGAS may be involved in EcSTING-mediated interferon immune responses. Next we investigated whether EccGAS interacts with EcSTING, EcTBK1, EcTAK1 and EcIRF3. Confocal microscopy results showed that the green fluorescence of pEGFP-EcSTING, pEGFP-EcTBK1, pEGFP-EcTAK1, and pEGFP-EcIRF3 colocalized with the red fluorescence of EccGAS (**Figure 7B**). We further verified the experimental results by CO-IP assay. The plamids of pcDNA3.1-EccGAS was co-transfected with pEGFP-EcSTING, pEGFP-EcTBK1, pEGFP-EcTAK1, pEGFP-EcIRF3, and pEGFP-C1, respectively, followed by immunoprecipitation (IP) and immunoblotting (IB) of whole cell lysates (WCLs). The results showed that EccGAS was detected in the IP products of EcSTING, EcTAK1, EcTBK1, and EcIRF3, but not in pEGFP-C1 (**Figures 7C–E**). Therefore, EccGAS inhibited EcSTING-mediated interferon immune response and interacted with EcSTING, EcTAK1, EcTBK1, and EcIRF3.

## 4 Discussion

Cytoplasmic DNA receptors, as part of the PRRs, play important roles in the innate immune response. One of the major cytoplasmic DNA receptors in humans is cGAS. It is homologous to oligoadenosine synthase, independent of nucleotide sequence, and thus able to recognize a variety of DNA viruses that invade the cytoplasm, including endogenous or threatening exogenous DNA (22–24). Currently, the function of cGAS is rarely reported in fish. In this study, a cGAS homolog from orange-spotted grouper *Epinephelus coioides* (EccGAS) was cloned and its role in the innate immune system during viral infection was studied. The results showed that cGAS is a negative regulator of STING-mediated IFN response in grouper.

In mammals, only one type of cGAS is typically found and plays an important role in the activation of type I IFN (25, 26). In fish, due to genome duplication events (27), two paralogs of cGAS were found in grass carp and zebrafish (28, 29). However, only one cGAS has been recorded in grouper. Several reports indicate that the C-terminus is highly conserved among cGAS homologs from fish to humans, whereas the N-terminal exhibits great diversity in length and sequence among the cGAS homologs (30). BLAST analysis showed that EccGAS had the highest similarity (71.8%) with *Sebastes umbrosus* and the lowest similarity with mammals. SMART prediction revealed that EccGAS has a highly conserved Mab21 domain, suggesting that it belongs to the MAB21 family. This domain was originally discovered in *Cryptobacterium showyeri* and plays a key role in DNA binding of cGAS (31).

Recently, mammalian cGAS was identified as a ubiquitous sensor of cytosolic dsDNA. However, the role of fish cGAS in innate immune regulation has not been elucidated. Previous studies reported that cGAS is widely expressed in different tissues. cGAS was highly expressed in the spleen and intestines of pigs and chickens (32, 33). cGAS was also highly expressed in the liver and intestine of grass carp (34). The liver and gut are the major sites of interferon-stimulated gene (ISG) expression during viral infection in fish (29); therefore, cGAS may play an important role in antiviral innate immunity in fish. In the present study, EccGAS was predominantly expressed in the blood, skin, and gills. Therefore, we speculate that the high expression of cGAS at these sites may be a strategy to balance the antiviral immune response to avoid over-induction of ISGs. After SGIV infection, the expression of EccGAS increased with the time of virus infection, suggesting that EccGAS is involved in the innate immune response. Similarly, grass carp cGASa was up-regulated under GCRV or poly dA:dT treatment (28), and grass carp cGASb was up-regulated under GCRV, poly dA:dT or poly I:C treatment (28, 35). Meanwhile, the Japanese medaka cGAS was significantly induced under *Edwardsiella tarda* treatment (36).

Studies have reported that cGAS activates the intrinsic antimicrobial defenses of cells and promotes autophagic targeting of *Mycobacterium tuberculosis* (37). Mice deficient in cGAS or STING exhibited lower type I IFN levels and higher viral loads (38). In crucian carp and grass carp, CacGAS and CicGAS reduced the CiRIG-I-mediated cellular antiviral response and promoted viral replication (39). After silencing EccGAS, the transcription levels of SGIV genes of MCP, ICP18, and VP19 were significantly inhibited, and the levels of MCP protein were increased, suggesting that EccGAS may promote SGIV replication. Knockdown of EccGAS potentiated the transcription of endogenous IFN1, IFN2, PKR, Viperin, ISG15, ISG56, IL-1β, IL-8, and TNFα in GS cells. EccGAS also inhibited the activation of IFN1, ISRE, and NF-κB promoters in GS cells. These results suggested that EccGAS is involved in the regulation of virus-induced IFN signaling.

Many studies have shown that cGAS is distributed in the cytoplasm of humans, pigs, chickens, and grass carp (11, 32–34). In our study, EccGAS and Mab21 were uniformly distributed in the cytoplasm, suggesting that grouper cGAS may have similar functions to mammalian cGAS. Several studies have shown that STING proteins in the endoplasmic reticulum were activated to recruit and bind with MAVS located in the mitochondria (10, 40); thus, they recruit TBK1 and activate IRF3 and NF-κB phosphorylation. In our previous study, EcSTING is distributed in the endoplasmic reticulum (41). In the present study, EccGAS is highly distributed in both the endoplasmic reticulum and mitochondria. Therefore, EccGAS may play an important role in the innate immune system by interacting with STING. In grass carp, cGASL interacts with STING and inhibits STING-mediated activation of gcIFN1pro (34). EccGAS can inhibit EcSTING-induced activation of IFN1, ISRE and NF-κB promoter activities. Furthermore, EcCGAS can colocalize with EcSTING. EccGAS and EcSTING can also interact, which is independent of the Mab21 domain. These results suggest that EccGAS could interact with STING and inhibit STING-mediated activation of IFN.

As important components of the cGAS-STING signaling pathway, EcTBK1, EcTAK1, and EcIRF3 are expressed in grouper and play important roles in antiviral innate immunity (42–44). We then investigated the roles of EccGAS in EcTBK1-, EcTAK1-, and EcIRF3-mediated activation of IFN. The results showed that EccGAS inhibited the activities of IFN1, ISRE and NF-κB promoters induced by EcTBK1, EcTAK1 and EcIRF3. Confocal microscopy showed that EcTBK1, EcTAK1, and EcIRF3 colocalized with EccGAS. Co-IP showed that EccGAS interacted with EcTBK1, EcTAK1, and EcIRF3. Thus, EccGAS inhibits STING-mediated interferon immune responses and interacts with STING, TAK1, TBK1, and IRF3.

In summary, a cGAS homolog of grouper (EccGAS), which contains a typical Mab21 structural domain, was identified for the first time. EccGAS was evenly distributed in the cytoplasm and partially co-located in the endoplasmic reticulum and mitochondria. EccGAS could promote SGIV replication. EccGAS negatively regulates EcSTING-mediated interferon immune responses and interacts with EcSTING, EcTAK1, EcTBK1, and EcIRF3. These findings contribute insights and methods for the prevention and treatment of viral infections.

## Data availability statement

The original contributions presented in the study are included in the article/supplementary material. Further inquiries can be directed to the corresponding authors.

## Ethics statement

The animal study was reviewed and approved by The Animal Care and Use Committee of College of Marine Sciences, South China Agricultural University.

## Author contributions

Conceptualization, JW and QQ. Methodology, LZ. Software, LZ. Validation, XZ, JL and LX. Investigation, SK, JL, HC, MS, STW, ZX, SW. Writing-original draft preparation, LZ. Writing-review and editing, JW, QQ. Supervision, QQ. Funding acquisition, QQ, JW, SW. All authors have read and agreed to the published version of the manuscript.
